# Evaluation and Management of Dyslipidemia in Patients Treated with Lorlatinib

**DOI:** 10.3390/curroncol28010029

**Published:** 2021-01-04

**Authors:** Normand Blais, Jean-Philippe Adam, John Nguyen, Jean C. Grégoire

**Affiliations:** 1 Service d’Hématologie-Oncologie, Département de Médicine, Centre Hospitalier de l’Université de Montréal (CHUM), Montréal, QC H2X 3E4, Canada; 2 Département de Pharmacie, Centre Hospitalier de l’Université de Montréal (CHUM), Université de Montréal, Montréal, QC H2X 3E4, Canada; jean-philippe.adam.chum@ssss.gouv.qc.ca (J.-P.A.); johnofnguyen@gmail.com (J.N.); 3 Département de Cardiologie, Institut de Cardiologie de Montréal, Montréal, QC H2X 3E4, Canada; J.Gregoire@icm-mhi.org

**Keywords:** non-small cell lung cancer, hypercholesterolemia, hypertriglyceridemia, ALK inhibitor, tyrosine kinase inhibitor

## Abstract

The use of lorlatinib, an anaplastic lymphoma kinase (ALK) inhibitor for the treatment of *ALK*-positive metastatic non-small cell lung cancer, is associated with dyslipidemia in over 80% of patients. Clinical trial protocols for the management of lorlatinib-associated dyslipidemia differ from clinical practice guidelines for the management of dyslipidemia to prevent cardiovascular disease, in that they are based on total cholesterol and triglyceride levels rather than on the low-density lipoprotein cholesterol and non-high-density lipoprotein cholesterol levels that form the basis of current cardiovascular guideline recommendations. In order to simplify and harmonize the management of cardiovascular risk in patients with lorlatinib, an advisory committee consisting of a medical oncologist, a cardiologist, and two pharmacists with expertise in cardiology and oncology aimed to develop a simplified algorithm, adapted from the Canadian Cardiovascular Society dyslipidemia recommendations. Recommendations for the evaluation and management of hypercholesterolemia and isolated hypertriglyceridemia in patients treated with lorlatinib are outlined. These recommendations are based on data collected in a large number of lipid-lowering therapy trials applicable to individuals with and without cancer. Considering the relatively long life expectancy and improving prognosis of patients with *ALK* translocations, this specific patient population should be treated as are patients without cancer and are likely to derive the same benefits from lipid-lowering therapy.

## 1. Introduction

For the approximately 0.5–3% of patients with non-small cell lung cancer (NSCLC) with rearrangements of the anaplastic lymphoma kinase *(ALK)* gene in Canada, standard therapy consists of a first- or second-generation ALK tyrosine kinase inhibitor (TKI), such as alectinib, brigatinib, crizotinib, or ceritinib [1]. However, most patients eventually develop secondary resistance to these therapies [2]. Lorlatinib is a third-generation ALK/ROS1 TKI that has demonstrated potency against most known resistance mutations to first- and second-generation ALK TKIs [3]. Lorlatinib is thus indicated for the treatment of *ALK*-positive metastatic NSCLC in patients who have progressed on first- and/or second-generation ALK inhibitors [4].

The efficacy and safety of lorlatinib were established in a phase 2 trial of 275 patients with *ALK1*-positive or *ROS1*-positive advanced NSCLC [5]. The most common treatment-related adverse events were hypercholesterolemia and hypertriglyceridemia, which occurred in 81% and 60% of patients, respectively. Grade 3–4 hypercholesterolemia and hypertriglyceridemia were each observed in 16% of patients [6]. Median time to onset of hyperlipidemia was 15 days (range 1–219 days) [6]. Despite the high prevalence of hypercholesterolemia and hyperlipidemias, these adverse events did not result in any treatment discontinuations and infrequently led to delayed or reduced doses of lorlatinib, with 3.4% and 4.7% of patients having delayed doses and 0.7% having reduced doses. 

In the context of the phase 2 trials, most hyperlipidemias were managed by the addition of lipid-lowering therapy, with 81% of patients receiving at least one lipid-lowering agent [6]. In this clinical trial, the management of hyperlipidemia was determined based on the Common Terminology Criteria for Adverse Events (CTCAE) classification, with lipid-lowering therapy being introduced or intensified for all grades of adverse events (i.e., cholesterol over the upper limit of normal (ULN) and/or triglycerides ≥ 1.71 mmol/L) [6,7]. In the event of grade 4 hyperlipidemia (defined as total cholesterol (TC) > 12.92 mmol/L, triglycerides (TG) > 11.4 mmol/L, and/or life-threatening consequences), lorlatinib was withheld until levels resolved to grade 2 or lower (TC ≤ 10.34 mmol/L and TG ≤ 5.7 mmol/L), a statin or other lipid-lowering therapy was initiated or optimized, and the lorlatinib dose was reduced by one dose level or rechallenged at the same dose.

The cancer clinical trial protocols for the management of hyperlipidemia differ from existing clinical practice guidelines for the management of dyslipidemia to prevent cardiovascular disease, in that they are based on TC and TG levels rather than on the low-density lipoprotein cholesterol (LDL-C) and non-high-density lipoprotein cholesterol (non-HDL-C) levels that form the basis of the American, Canadian, and European cardiovascular guideline recommendations [8,9,10]. 

In order to simplify and harmonize the management of cardiovascular risk in patients receiving lorlatinib treatment, an advisory committee consisting of a medical oncologist, a cardiologist, and two pharmacists with expertise in cardiology and oncology aimed to develop a simplified algorithm, adapted from the Canadian Cardiovascular Society (CCS) recommendations [8]. 

## 2. Discussion

The proposed algorithm, adapted from the CCS recommendations [8], is outlined in Table 1. Additionally, as these guidelines do not specifically address isolated hypertriglyceridemia, a condition that may potentially be caused by lorlatinib treatment, the management of isolated hypertriglyceridemia is addressed separately in Table 2. 

### 2.1. Evaluation of Cardiovascular Risk

As indicated in Table 1 and Table 2, the first step in managing dyslipidemia in patients treated with lorlatinib is to evaluate their level of cardiovascular risk. Risk evaluation of these patients is particularly relevant given the lorlatinib-associated hyperlipidemia and the relatively long life expectancy of NSCLC patients treated with ALK inhibitors. The main goals of this evaluation are i) to understand why a patient may be taking statin therapy before the initiation of lorlatinib, ii) to determine whether initiation or intensification of lipid-lowering therapy is required, and iii) to select an appropriate statin dose if needed. This advisory committee has adopted a simplified approach to evaluating cardiovascular risk in order to enable rapid assessment by the oncologist. Instead of mandating the calculation of Framingham Risk Scores, as recommended by the CCS, we suggest evaluating cardiovascular risk based on lipid levels and the presence or absence of statin-indicated conditions, including a history of one of the following: Stroke, Transient ischemic attack, Abdominal aortic aneurysm, Myocardial infarction, Peripheral vascular disease, Chronic kidney disease, or Diabetes (as described by the STAMP-CD mnemonic). 

### 2.2. Monitoring of Lipid Parameters

Because of the high incidence of hypercholesterolemia and hypertriglyceridemia shortly after the initiation of lorlatinib treatment, we recommend ordering a complete lipid profile at baseline, at 1, 2 and 3 months after starting therapy, and every 3 months thereafter. The complete lipid profile should include measurement of TC, LDL-C, HDL-C, and TG. Non-HDL-C levels may be calculated as the difference between TC and HDL-C. It should be noted that there is no need for the patient to be fasting in order for this complete lipid profile to be measured and therefore these tests can therefore be performed at any time of the day. However, if TG levels are >4.5 mmol/L, a fasting lipid profile should be done. The close monitoring of lipid parameters, as suggested in Table 1 and Table 2, enables the rapid initiation of lipid-lowering therapy, as soon as after one month of lorlatinib treatment, as well as titration of the lipid-lowering therapy at monthly intervals until the target is reached. Additionally, this monitoring is in accordance with the usual timing of other laboratory tests (e.g., complete blood count and biochemistry) that should be done to monitor lorlatinib therapy. 

### 2.3. Initiation and Titration of Lipid-Lowering Therapy

As previously discussed, contrary to the protocol established by the phase 2 study of lorlatinib, TC itself is not recommended as a criterion for the initiation of a lipid-lowering therapy according to the CCS and other current guidelines. Instead, in the event of hyperlipidemia secondary to lorlatinib therapy as determined by LDL-C ≥ 3.5 mmol/L, non-HDL-C level ≥ 4.3 mmol/L, or TG > 5.7 mmol/L, the expert group recommends the initiation of lipid-lowering therapy in order to avoid the need for interruption or dose adjustments of lorlatinib treatment. Additionally, all secondary prevention patients treated with lorlatinib should also be treated with appropriate statin therapy. 

Based on their proven efficacy and low involvement with the specific CYP450 enzymes responsible for the metabolism of lorlatinib (i.e., CYP3A), rosuvastatin and pravastatin are recommended as first-line lipid-lowering therapy in patients treated with lorlatinib. The starting dose of these statins should be determined based on the patient’s baseline cardiovascular risk level. Primary prevention patients may be started on pravastatin 20 mg once daily or rosuvastatin 5 mg once daily, while secondary prevention patients should be initiated on pravastatin 40 mg once daily or rosuvastatin 20 to 40 mg once daily. Secondary prevention patients who are being treated with other statins should be switched to equivalent doses of pravastatin or rosuvastatin in order to avoid potential drug–drug interactions. Statin therapy is expected to reduce LDL-C levels by 20–60% and TG levels by 5–30% [11,12]. Statin dosage should be adjusted as necessary until the lipid target is reached. For patients with hypercholesterolemia, the lipid targets are those suggested by the CCS guidelines as outlined in Table 1, while for patients with isolated hypertriglyceridemia, a target of TG ≤ 11.4 mmol/L has been suggested in order to obviate the need to withhold lorlatinib.

If lipid targets are not met with an optimized dose of statin therapy, referral to a lipid specialist should be considered. Alternatively, the addition of a second-line lipid-lowering agent, such as ezetimibe 10 mg once daily or fenofibrate 200 mg once daily, may also be considered based on the clinical situation. These add-on therapies are expected to reduce LDL-C levels by an additional 5–25% and TG levels by 5–50% [13,14]. The addition of omega-3 fatty acids of marine origin at a dose of 2–4 g/day may also be considered to reduce TG levels by an additional 25–35% [15].

### 2.4. Lifestyle Modification Strategies

We also suggest that all patients on lorlatinib requiring statin therapy be referred for multidisciplinary cardiovascular risk management by a nutritionist, primary care physician, and/or other healthcare providers, as dietary intervention can be an important tool in the management of lipid levels and control of other cardiovascular risk factors, such as diabetes, hypertension, obesity, physical inactivity, and active smoking, are important for management of overall cardiovascular risk. 

### 2.5. Lorlatinib Management in Patients with Hyperlipidemia

Lorlatinib dosage should not be modified for most patients who experience hyperlipidemia secondary to treatment. Clinicians may consider temporarily withholding lorlatinib therapy in patients who experience grade 4 hyperlipidemia (defined as TG > 11.4 mmol/L, TC > 12.92 mmol/L, and/or life-threatening consequences) until TG levels are reduced to ≤11.4 mmol/L. Additionally, dose reduction of lorlatinib may be considered in patients for whom grade 4 hyperlipidemia reoccurs despite optimal pharmacological and non-pharmacological therapy. 

### 2.6. Ongoing Management

For most patients for whom statins are required, lipid-lowering therapy should be continued as long as the patient remains on lorlatinib. Once lorlatinib therapy has been discontinued, the lipid-lowering therapy should be re-evaluated and either discontinued or reduced to pre-lorlatinib levels, although lipid levels should continue to be monitored. Additionally, based on clinical judgment, clinicians may consider discontinuing lipid-lowering therapy in palliative patients with a limited life expectancy (e.g., less than one year). 

## 3. Conclusions

This panel recommends a simple algorithm to diagnose and manage dyslipidemia in patients undergoing therapy with lorlatinib in line with CCS guidelines applicable to the general population. These guidelines are based on data collected in a large number of lipid-lowering therapy trials applicable to individuals with and without cancer. Considering the relatively long life expectancy and improving prognosis of patients with *ALK* and *ROS1* translocations, this panel feels that this specific patient population should be treated as are patients without cancer and are likely to derive the same benefits from lipid-lowering therapy.

## Figures and Tables

**Table 1:** Recommendations for the evaluation and management of hypercholesterolemia in patients treated with lorlatinib.

Risk Stratification	Criteria to Initiate Lipid-Lowering Therapy	Target	Management of Lorlatinib	First-Line Therapy	Second-Line Therapy ^‡^	Monitoring and Follow-Up
**Primary Prevention**	LDL-C ≥ 3.5 mmol/L or non-HDL-C ≥ 4.3 mmol/L	LDL-C < 2.0 mmol/L or ↓ LDL-C > 50% from pre-treatment levels or non-HDL-C < 2.6 mmol/L	If TC ≤ 12.92 mmol/L, continue lorlatinib at the same dose If TC > 12.92 mmol/L, withhold lorlatinib until TC ≤ 12.92 mmol/L, and then restart lorlatinib at the previous dose	Pravastatin 20 mg once daily **^†^ (max: 80 mg) or Rosuvastatin 5 mg once daily ^†^ (max: 40 mg)	Ezetimibe 10 mg once daily ^¶^	Measure complete lipid profile at baseline, at 1, 2, and 3 months after starting therapy, and every 3 months thereafter
Secondary Prevention *	STAMP-CD			Pravastatin 40 mg once daily **^†^ (max: 80 mg) or Rosuvastatin 20–40 mg once daily ^†^		

* Statin-indicated conditions. ** Decrease dose by 50% in patients with significant renal or hepatic dysfunction. ^†^ Statin therapy is expected to reduce LDL-C levels by 20–60% and TG levels by 5–30%. ^‡^ If target is not met with optimized dose of statin as first-line therapy, consider referral to lipid specialist for addition of second-line therapy. ^¶^ Ezetimibe is expected to reduce LDL-C levels by 14–25% and TG levels by 7–9%. HDL-C: high-density lipoprotein cholesterol; LDL-C: low-density lipoprotein cholesterol; STAMP-CD: Stroke, Transient ischemic attack, Abdominal aortic aneurysm, Myocardial infarction, Peripheral vascular disease, Chronic kidney disease, Diabetes; TC: total cholesterol.

**Table 2:** Recommendations for the evaluation and management of isolated hypertriglyceridemia in patients treated with lorlatinib.

Risk Stratification	Criteria to Initiate Lipid-Lowering Therapy	Target	Management of Lorlatinib	First-Line Therapy	Second-Line Therapy ^‡^	Monitoring and Follow-Up
**Primary Prevention**	TG > 5.7 mmol/L and/or life-threatening consequences	Achieve and maintain TG ≤ 11.4 mmol/L (to avoid dose interruptions of lorlatinib)	If TG ≤ 11.4 mmol/L, continue lorlatinib at the same dose If TG > 11.4 mmol/L, withhold lorlatinib until TG ≤ 11.4 mmol/L, and then restart lorlatinib at the previous dose	Pravastatin 20 mg once daily **^†^ (max: 80 mg) or Rosuvastatin 5 mg once daily ^†^ (max: 40 mg)	Fenofibrate 200 mg once daily ^¶^ and/or omega-3 fatty acids 2–4 g/day ^¶¶^	Measure complete lipid profile at baseline, at 1, 2, and 3 months after starting therapy, and every 3 months thereafter Monitor amylase/lipase and signs of pancreatitis
**Secondary** Prevention *				Pravastatin 40 mg once daily **^†^ (max: 80 mg) or Rosuvastatin 20-40 mg once daily ^†^		

* Statin-indicated conditions. ** Decrease dose by 50% in patients with significant renal or hepatic dysfunction. ^†^ Statin therapy is expected to reduce LDL-C levels by 20–60% and TG levels by 5–30%. ^‡^ If target is not met with optimized dose of statin as first-line therapy, consider referral to lipid specialist for addition of second-line therapy. ^¶^ Fenofibrate is expected to reduce LDL-C levels by 5–20% and TG levels by 20–50%. ^¶¶^ Omega-3 fatty acids of marine origin are expected to reduce TG levels by 25–35%. LDL-C: low-density lipoprotein cholesterol; TG: triglyceride.
